# The microbial biosynthesis of noncanonical terpenoids

**DOI:** 10.1007/s00253-024-13048-y

**Published:** 2024-02-21

**Authors:** Mengyu Ma, Mingkai Li, Zhenke Wu, Xiqin Liang, Qiusheng Zheng, Defang Li, Guoli Wang, Tianyue An

**Affiliations:** Featured Laboratory for Biosynthesis and Target Discovery of Active Components of Traditional Chinese Medicine, School of Integrated Traditional Chinese and Western Medicine, Binzhou Medical University, Yantai, 264003 China

**Keywords:** Terpenoids, Biosynthesis, Five carbon units, Noncanonical terpenoids, Carbon skeleton

## Abstract

**Abstract:**

Terpenoids are a class of structurally complex, naturally occurring compounds found predominantly in plant, animal, and microorganism secondary metabolites. Classical terpenoids typically have carbon atoms in multiples of five and follow well-defined carbon skeletons, whereas noncanonical terpenoids deviate from these patterns. These noncanonical terpenoids often result from the methyltransferase-catalyzed methylation modification of substrate units, leading to irregular carbon skeletons. In this comprehensive review, various activities and applications of these noncanonical terpenes have been summarized. Importantly, the review delves into the biosynthetic pathways of noncanonical terpenes, including those with C6, C7, C11, C12, and C16 carbon skeletons, in bacteria and fungi host. It also covers noncanonical triterpenes synthesized from non-squalene substrates and nortriterpenes in *Ganoderma lucidum*, providing detailed examples to elucidate the intricate biosynthetic processes involved. Finally, the review outlines the potential future applications of noncanonical terpenoids. In conclusion, the insights gathered from this review provide a reference for understanding the biosynthesis of these noncanonical terpenes and pave the way for the discovery of additional unique and novel noncanonical terpenes.

**Key points:**

•*The activities and applications of noncanonical terpenoids are introduced*.

•*The noncanonical terpenoids with irregular carbon skeletons are presented*.

•*The microbial biosynthesis of noncanonical terpenoids is summarized*.

## Introduction

Terpenoids are a large group of structurally complex natural products widely distributed in nature, with a predominant presence in the secondary metabolites of plant, animal, and microoganism. The biosynthesis of terpenoids is a highly intricate process. To date, over 90,000 terpenoids have been identified, primarily originating from plants, microorganisms, and marine organisms (Huang et al. 2022; Li et al. 2023b; Ma et al. 2023a). Terpenoids exhibit a wide array of unique physicochemical properties and biological activities, making them indispensable in various industries such as medicine, cosmetics, food processing, biofuels, and more. They hold substantial economic value and broad market prospects (Li et al. 2020; Song et al. 2023; Zhu et al. 2021). Terpenoids encompass common subclasses like monoterpenes, sesquiterpenes, diterpenes, and triterpenes. Monoterpenes, characterized by their relatively smaller molecular weight, include oxygenated derivatives known for their potent aroma and biological effects. Some monoterpenoids, such as carvacrol, menthol, and citronellol, exhibit antimicrobial, antiviral, antioxidant, and anticancer properties (Kamatou et al. 2013; Sharifi-Rad et al. 2018; Silva et al. 2020; Singh et al. 2023). These compounds serve as crucial raw materials in pharmaceuticals, food, and cosmetics (Zhu et al. 2021). Sesquiterpenoids, the most prevalent group among terpenoids, and their oxygenated derivatives are often found in volatile oils, contributing to strong aroma and remarkable biological activities (Guo et al. 2021). For instance, as a constituent of the volatile oil of ginseng, a bicyclic sesquiterpene, β-caryophyllene possesses a variety of pharmacological activities such as anti-inflammatory (Scandiffio et al. 2020), neuroprotective (Hu et al. 2022), and immunomodulatory (Baradaran Rahimi and Askari 2022). Another notable sesquiterpene containing an intracyclic peroxy group, artemisinin, extracted from *Artemisia annua* L., is the most potent antimalarial drug (Ma et al. 2020). Diterpenoids, such as gibberellins (GAs), play vital in primary plant metabolism, growth, and development (Yamaguchi 2008). They also possess great industrial value as biofuels, food additives, or perfume ingredients (Ren et al. 2022). On the other hand, triterpenoids are the most common nonsteroidal secondary metabolites in terrestrial and marine plants and animals. Tetracyclic and pentacyclic triterpenoids are the most prevalent in this category (Stonik and Kolesnikova 2021), including compounds such as oleanolic acid (Castellano et al. 2022), betulinic acid (Jiang et al. 2021), and ginsenoside (Li et al. 2023a). They exhibit high pharmacological activity in various aspects such as antitumor effects, lipid regulation, hepatoprotection, and immunomodulation. Due to their important activities, lots of valuable terpenoids have been derived by reconstruction their biosynthetic pathways in microbial cell factories (An et al. 2020; Ma et al. 2023b; Zha et al. 2020).

Terpenoids, which are natural products based on the C5 isoprene unit, undergo a biosynthesis process that can be broadly divided into four steps: building block formation, condensation, core structure assembly, and post-modification (Xiao et al. 2019). These steps involve the generation of the C5 skeletal units, condensation of these units to form larger precursors, the assembly of these precursors into terpenoids, and the subsequent modification of these terpenoids. The biosynthesis begins with the generation of the C5 skeletal units, isopentenyl diphosphate (IPP) and 3,3-dimethylallyl diphosphate (DMAPP). Despite the structural diversity of terpenoids, their biosynthesis consistently initiates with IPP and DMAPP, both of which are generated in nature via two main pathways: the methylerythritol phosphate (MEP) pathway and the mevalonate (MVA) pathway, representing the initial stages of terpenoid synthesis (Chen et al. 2021b; Tong et al. 2021). The MVA pathway is primarily found in eukaryotes, including yeast, higher plants and animals, and some archaea. However, the MEP pathway is present in some prokaryotes, cyanobacteria, and higher plants (Vranová et al. 2013). In plant plastids, IPP and DMAPP are synthesized via the MEP pathway, while in the cytoplasm, they are produced through the MVA pathway (Liao et al. 2016).

The MVA pathway begins with acetyl-coenzyme A (acetyl-CoA), and this compound is enzymatically transformed into 3-hydroxy-3-methylglutaryl CoA (HMG-CoA) through the actions of acetyl-CoA C-acetyl transferase (AACT) and 3-hydroxy-3-methylglutaryl CoA synthase (HMGS). Subsequently, this product is reduced by HMG-CoA reductase (HMGR) to generate MVA, which then undergoes pyrophosphorylation and decarboxylation to generate IPP. Afterward, isopentenyl pyrophosphate isomerase (IPPI) catalyzes the isomerization of IPP to DMAPP. In contrast, the MEP pathway starts with pyruvate and glyceraldehyde-3-phosphate, which are converted to IPP, proceeding through a series of seven enzymatic reactions involving enzymes such as 1-deoxy-D-xylulose-5-phosphate synthase (DXS), 1-deoxy-D-xylulose-5-phosphatereductoisomerase (DXR), 2-C-methyl-D-erythritol4-phosphatecytidylyltransferase (MCT), 4-diphosphocytidyl-2-C-metllyl-D-erythritolkinase (CMK), 2-C-methyl-D-erythritol-2,4-cyclodiphosphatesynthase (MDS), 4-hydroxy-3-methylbut-2-enyldiphosphatesynthase (HDS), and 4-hydroxy-3-methylbut-2-enyldiphosphatereductase (HDR).

In the second step of terpenoid synthesis, DMAPP and IPP are condensed by geranyl pyrophosphate synthase (GPPS) to produce GPP. The binding of GPP with another IPP results in the formation of farnesyl pyrophosphate (FPP). Further binding of IPP generates geranylgeranyl pyrophosphate (GGPP), and further condensation of two FPP forms squalene in the presence of squalene synthase (SS). These compounds serve as precursors to generate specific terpenes (Xiao et al. 2019). The third step involves the cyclization of these direct precursors by various terpene synthases (TPSs), assembling them into the core structures of different terpenes (Pazouki and Niinemets 2016; Wang et al. 2022). Finally, these terpene structures are catalyzed by various modifying enzymes such as cytochrome P450 enzymes (CYP450) and glycosyltransferases (GTs) to generate a variety of terpenes, significantly expanding the diversity within the terpenoid class (Couillaud et al. 2022).

The terpenoids, generated with the C5 unit as the backbone, adhere to the isoprene rule and are referred to as classical terpenoids (Ferguson et al. 1959; Ruzicka 1953). However, there exists a class of terpenoids characterized by several carbon atoms that do not align with multiples of five. Moreover, their terpene carbon skeleton is altered, such as methyltransferase-catalyzed methylation modifications of the substrate unit, forming terpenoids with irregular carbon skeletons, such as C6, C7, C11, C12, and C16. Such compounds are called noncanonical terpenoids (Sommer et al. 2022; Xia et al. 2022). In addition, non-squalene precursors have been identified in the biosynthesis of triterpenes.

The emergence of these noncanonical terpenoids and the discovery of corresponding TPSs have substantially diversified the terpene landscape. This expansion not only provides valuable insights into the natural synthesis of diverse terpenoids through structural and mechanistic characterization but also offers new opportunities for the exploration of novel terpenoid natural products and gene clusters. Recent advances in genomics, proteomics, transcriptomics, and other analytical techniques, along with progress in biosynthetic technologies, have deepened our knowledge of biosynthetic pathways for noncanonical terpenoids. Both rational and non-rational protein engineering approaches allow for the modification of enzyme activities and functions, leading to the discovery of additional noncanonical TPSs. The discovery of each new enzyme will further increase the diversity of terpenoids. These advances pave the way for the use of microbial cell factories to reconstruct biosynthetic pathways of noncanonical compounds, to produce downstream products with more complex structures.

This review provides a comprehensive summary of the activities and applications of noncanonical terpenoids. It also for the first time systemically summarized the microbial biosynthetic pathways for specific noncanonical terpenes, including C6, C7, C11, C12, and C16, as well as noncanonical triterpenes synthesized using non-squalene substrates and nortriterpenes in *Ganoderma lucidum*, which are outlined in detail. Finally, the future application prospects of noncanonical terpenoids are envisioned. In summary, this review provides a reference for the biosynthesis studies of these noncanonical terpenoids and the discovery of more novel noncanonical terpenoids.

## Activities of noncanonical terpenoids and their applications

### Pharmacological effects of noncanonical terpenoids

Numerous noncanonical terpenoids derived from medicinal plants have demonstrated various pharmacological effects such as antitumor, anti-inflammatory, and antioxidant properties. For instance, liguhodgcins A, a rare monoterpene compound with a unique δ-lactone-containing skeleton and a chlorine atom extracted from the Chinese medicinal plant *Ligularia hodgsonii*, showed positive responses against human leukemia cells (HL-60), human liver cancer cells (SMMC-7721), and human cervical cancer cells (HeLa) as indicated by the Sulforhodamine B (SRB) colorimetric assay (Chen et al. 2012). Moreover, among terpenes containing a non-regular carbon atom skeleton, the C16 terpene nor-iridal triterpenes iriwattins A from *Iris wattii* Baker exhibited significant inhibition of the enzyme protein tyrosine phosphatase 1B (PTP1B), with an inhibition rate of 52% (Ni et al. 2019). In addition, two C19 backbone-containing norterpenoids, cyclobutastellettolides A and B, belonging to isomalabaricanes were identified in *Stelletta* sp. marine sponge. Cytometric assays confirmed that they significantly enhanced reactive oxygen species (ROS) production in mouse macrophages (Kolesnikova et al. 2019).

### Agricultural applications of noncanonical terpenoids

In the realm of agriculture, noncanonical terpenoids have found applications in protecting crops from insects. Homoterpene, a common volatile terpene, is released by plants when attacked by herbivores as a means of recruiting their natural enemies for indirect defense. C16-homoterpene (*E,E*)-4,8,12-trimethyltrideca-1,3,7,11-tetraene (TMTT) and its C11 analog (*E*)-4,8- dimethyl-1,3,7-nonatriene (DMNT) are the two major homoterpenes (Lee et al. 2010). DMNT has shown significant resistance to *Plutella xylostella*, a common pest. As a natural product synthesized by plants, DMNT offers a safer alternative to chemically synthesized pesticides. It disrupts midgut microbiota populations, which are essential for DMNT-induced pest control, and represses the PxMucin gene in midgut cells, leading to peritrophic matrix rupture and subsequent death of *P. xylostella* (Chen et al. 2021a).

### Application of noncanonical terpenoids in biofuels

Noncanonical terpenes, distinguished by their unique carbon compositions compared to classical terpenes, exhibit distinct properties and have found applications in various fields. Fuel performance tests have demonstrated the potential of C6-isoprenol and C7-isoprenol compounds, produced using isoprenol as a substrate. They have lower water solubility and similar or higher energy densities compared to classical terpenes. In addition, C6-isoprenol and C7-isoprenol have comparable research octane number (RON) enhancement effects on isoprenol. Therefore, these two chemicals can be potential blends for gasoline and strong candidates for next-generation biofuels (Pang et al. 2021).

### Other applications of noncanonical terpenoids

Geosmin and 2-methylisoborneol (2-MIB) are the main compounds responsible for earthy-musty odor in water. As a C11 terpenoid, 2-MIB is a methylated monoterpene (Bentley and Meganathan 1981; Dickschat et al. 2007; Zaroubi et al. 2022) and was first discovered in streptomycete. Subsequently, it was detected in a variety of microorganisms such as actinomycetes, myxobacteria, and cyanobacteria (Rabe et al. 2013). 2-MIB imparts an earthy aromatic flavor to Brie and Camembert cheeses (Ignea et al. 2018).

Similar to 2-MIB, geosmin was first identified and isolated from streptomycetes and has a soil-specific odor (Dickschat 2016; Jüttner and Watson 2007). Geosmin can serve as a warning signal to indicate toxin production by microorganisms, thus deterring predation by eukaryotes. In a predation experiment, geosmin reduced predation on *Streptomyces coelicolor* by *Caenorhabditis elegans*, thereby reducing *C. elegans* mortality. This experiment demonstrated that the odor signal from geosmin is beneficial to both predators and prey (Zaroubi et al. 2022). Most geosmin and 2-MIB in water are produced by cyanobacteria (Giglio et al. 2008). Therefore, an in-depth study of the nature and biosynthesis of geosmin and 2-MIB could be useful in addressing odor-related issues in water resources.

On the other hand, volatile compounds released by the rhizobium *Serratia plymuthica* 4Rx13 inhibit plant and bacterial growth. Sodorifen, a C16 sesquiterpene, produced by this bacterial strain, is responsible for growth inhibition (von Reuss et al. 2018). Moreover, sodorifen is an important molecule involved in long-distance communication between fungi and bacteria, highlighting its significance in long-distance communication between fungi and bacteria (Schmidt et al. 2017).

## Microbial biosynthesis of noncanonical terpenoids

### Biosynthesis of noncanonical terpenoids with C6 and C7 carbon skeletons

An S-adenosyl-methionine (SAM)-dependent IPP methyltransferase (IPPMT), discovered in *Streptomyces monomycini*, can synthesize a variety of C6 and C7 prenyl pyrophosphates in vitro using IPP as a substrate. These prenyl pyrophosphates include the major product C6 compound (*E*)-4-methyl-IPP, the minor products (*Z)*-4-methyl-IPP and 4-methyl-DMAPP, as well as the C7 compounds, that is, 4,4-dimethyl-IPP and 4,4-dimethyl-DMAPP. The synthesis pathways of C6 and C7 compounds involve a series of enzymatic steps. The double bond formation between C3 and C4 of IPP is catalyzed by IPPMT, resulting in an additional methyl group attached to the C4 position to form a carbonium ion intermediate. The C6 compounds, such as 4-methyl-IPP, are generated by deprotonation of the C4 atom. The C7 compounds are derived from the C6 compounds through a second methylation. Adding another methyl group between the C3 and C4 positions connected to the C4 position of the C6 compound can form a carbocation. Finally, the two C atoms adjacent to C3 form the corresponding C7 compounds by deprotonation, respectively. In this process, the methyl group of SAM is transferred to the corresponding substrate via a SAM-dependent methyltransferase (SAM-MT), resulting in the synthesis of the corresponding prenyl pyrophosphate (Drummond et al. 2019). The methyltransferases MT1 and MT2 from *Rhodococcus fascians* can catalyze different methylation reactions using IPP as a substrate. In the presence of MT1, IPP generates the C6 compounds Me-IPP. Subsequently, Me-IPP undergoes a second methylation modification in the presence of MT2, resulting in the formation of the C7 compound dimethylated IPP (Radhika et al. 2015).

IPPMT selectively employs IPP as a substrate to generate methylation products and does not utilize DMAPP as a preferred substrate. Similarly, MT1 and MT2 do not efficiently use DMAPP as a substrate. However, homology modeling and ligand docking along with modification of GPP C2-methyltransferase (GPPC2MT) from *S. coelicolor* by site-saturation mutagenesis yield the mutant G202M, which efficiently uses DMAPP as a substrate to generate 2-methyl-DMAPP. Furthermore, G202M was identified as a key amino acid residue site for converting substrate specificity (Xia et al. 2022). In *Micromonospora humi*, a methyltransferase, humMT, has been identified. HumMT can react with SAM and IPP in vitro to produce the C6 compound 5-methyl-isoprenol. Additionally, it reacts with DMAPP to undergo a methylation modification at the C2 position, thereby producing a dephosphorylated product of 2-methyl-IPP, 2-methyl-isoprenol (Drummond et al. 2022). Furthermore, a gene, lon23, identified from *S. argenteolus* sharing 38% homology in amino acid sequence with GPPMT identified from *S. coelicolor*, could catalyze the formation of 3*Z*-3-methyl IPP (*Z*-homo-IPP) in the presence of Mg^2+^ and SAM using IPP as a substrate (Ozaki et al. 2018).

**Fig. 1:**
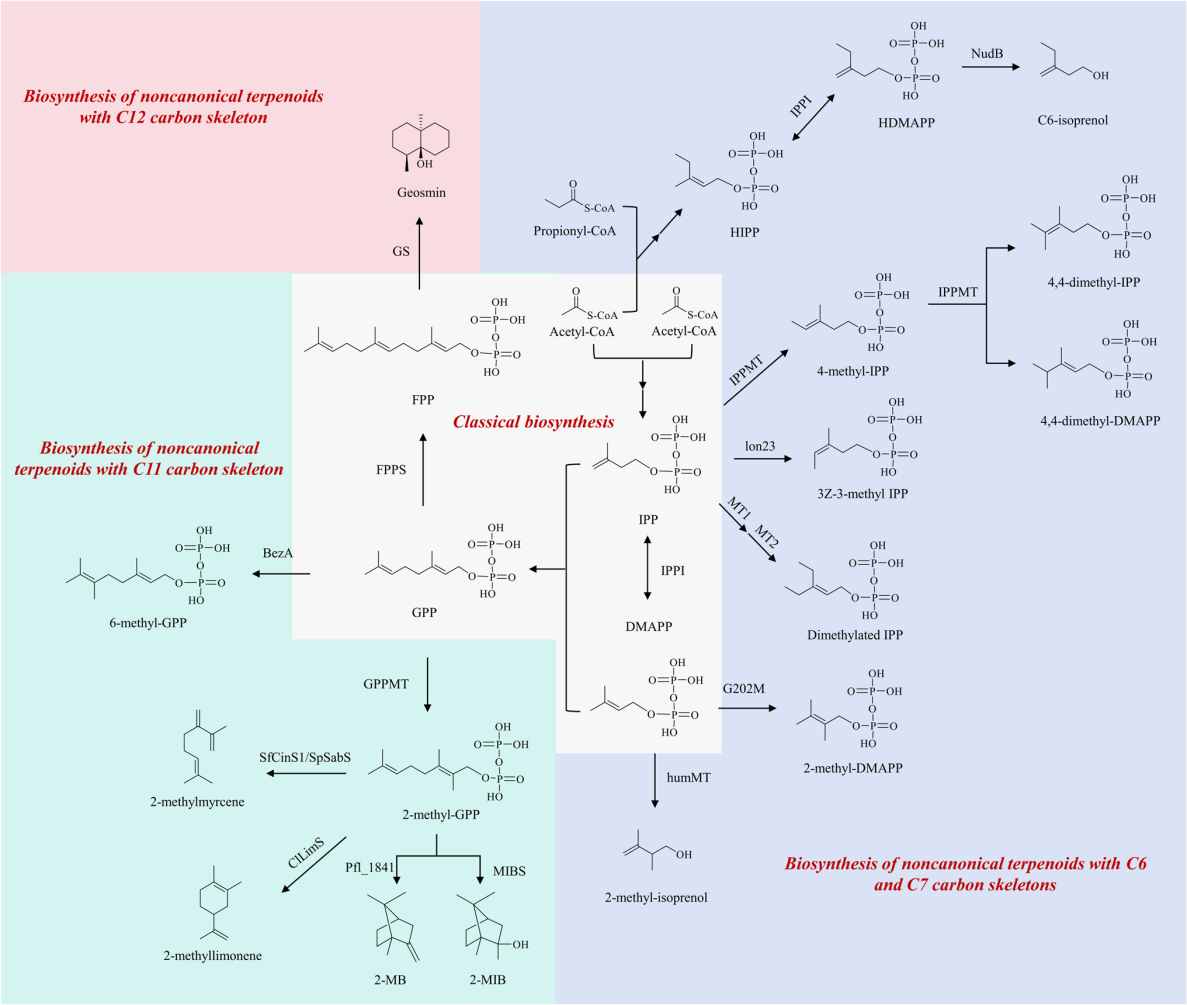
The biosynthesis of noncanonical terpenoids with C6, C7, C11, and C12 carbon skeletons. IPP isopentenyl diphosphate, DMAPP 3,3-dimethylallyl diphosphate, GPP geranyl pyrophosphate, FPP farnesyl pyrophosphate, IPPI IPP isomerase, HIPP homoisopentenyl pyrophosphate, HDMAPP homodimethylallyl pyrophosphate, NudB promiscuous phosphatase, IPPMT IPP methyltransferase, lon23 methyltransferase, MT1 methyltransferase from *Rhodococcus fascians*, MT2 methyltransferase from *Rhodococcus fascians*, G202M GPP C2-methyltransferase mutant from *S. coelicolor*, HumMT WP_091072690 from *M. humi*, BezA GPP C6-methyltransferase, FPPS FPP synthase, GS geosmin synthase, GPPMT GPP methyltransferase, 2-MB 2-methylenebornane, 2-MIB 2-methylisoborneol, MIBS 2-MIB synthase, Pfl_1841 2-MB synthase, ClLimS limonene synthase from *C. limon*, SfCinS1 1,8-cineole synthase from *S. fruticosa*, SpSabS sabinene synthase from *S. pomifera*

The synthesis of common terpenoids commences with the condensation of two acetyl-CoA molecules to form acetoacetyl-CoA. In contrast to this classical biosynthetic pathway, the lepidopteran mevalonate (LMVA) biosynthetic pathway in Lepidoptera begins with the condensation of propionyl-CoA and acetyl-CoA. This process results in the generation of C6 terpenes, specifically homoisopentenyl pyrophosphate (HIPP) and homodimethylallyl pyrophosphate (HDMAPP) (Eiben et al. 2019). By optimizing the LMVA pathway and introducing promiscuous phosphatase NudB, it is possible to establish a biosynthetic pathway for C6 compounds such as 3-ethyl-3-buten-1-ol (C6-isoprenol) in *E. coli*. Further introduction of PcCL and MlD genes to redirect the thiolase-dependent pathway from the upstream part of the LMVA pathway to the β-oxidation pathway and knockdown of the endogenous thiolase gene of *E. coli* could enhance HIPP yield, thus generating a large amount of C6-isoprenol under the catalytic effect of NudB (Pang et al. 2021). Figure 1 summarized the biosynthesis of the above noncanonical terpenoids with C6 and C7 carbon skeletons.

### Biosynthesis of noncanonical terpenoids with C11 and C12 carbon skeleton

#### Biosynthesis of 2-methyl-GPP and 6-methyl-GPP

GPP methyltransferase (GPPMT) from *S. coelicolor* A3(2) was the first methyltransferase found to produce 2-methyl-GPP. The structural analysis of GPPMT using structural biology revealed its catalytic mechanism (Köksal et al. 2012a, 2012b). In addition, the crystal structure of GPPMT from *S. lasaliensis* was further resolved, in which most of the methylation reactions were catalyzed by SAM-MTs (Ariyawutthiphan et al. 2011).

After introducing the GPPMT gene PlGPPMT and the 2-MIB synthase (MIBS) gene PlMIBS from *Pseudanabaena limnetica* into *Saccharomyces cerevisiae*, the engineered yeast strains produced major products such as 2-methylenebornane (2-MB), 1-methylcamphene (1MC) and 2-methyl-2-bornene (2M2B), along with a small number of minor products with a C11 structure, such as 2-methylmyrcene, 2-methyllimonene, 2-methyl linalool, and 2-methyl-α-terpineol. When 2-methyl-GPP was used as a precursor for generating noncanonical terpenes, the introduction of Erg20pN127W, a mutant of the bifunctional enzyme Erg20p, along with its fusion with the mutant PlGPPMTF266H protein, further increased the 2-methyl-GPP yield. Subsequently, classical TPSs of plant origin, including 1,8-cineole synthase (SfCinS1) from *Salvia fruticosa*, sabinene synthase (SpSabS) from *S. pomifera*, pinene synthase (PtPinS) from *Pinus taeda*, or limonene synthase (ClLimS) from *Citrus limon*, were introduced. C11 compounds were detected in all products. Among the yeast-producing 2-methyl-GPP, the major C11 products of SfCinS1 and SpSabS were 2 MB and 2-methylmyrcene, and the major C11 product of ClLimS was 2-methyllimonene. Thus, classical TPSs could use 2-methyl-GPP as a precursor to generate multiple C11 compounds (Ignea et al. 2018).

The substrate of GPPMT contains a non-conjugated olefin, and its reaction mechanism is similar to that of the steroid methyltransferase (SMT) family. Both GPPMT and SMT share a conserved glutamic acid residue. The GPPMT-mediated methylation reaction is likely initiated by the electrophilic attack of SAM on the C2 and C3 double bonds of GPP, leading to the formation of a carbonium ion intermediate at C3. Subsequently, 2-methyl-GPP is produced through deprotonation (Ariyawutthiphan et al. 2012).

In addition to GPPMT at the C2 position, a new GPPMT at the C6 position called BezA was identified in *Streptomyces argenteolus*. BezA can undergo a methylation modification reaction at the C6 position of the GPP to produce 6-methyl-GPP in the presence of Mg^2+^ and SAM in vitro (Tsutsumi et al. 2018, 2022).

#### Biosynthesis of 2-MIB and 2 MB

The biosynthetic pathway of 2-MIB in various bacteria, including actinomycete, myxobacterium, and *Pseudomonas fluorescens*, has been elucidated through isotope-labeled substrate feeding and in vitro synthesis using recombinant enzymes. Using SAM as a methyl donor, GPP undergoes a methylation modification reaction at the C2 position to generate 2-methyl-GPP catalyzed by SAM-dependent GPPMT. Subsequently, 2-MIB was generated by cyclization of MIBS (Chou et al. 2011; Dickschat et al. 2007; Komatsu et al. 2008; Wang and Cane 2008) (Fig. 1). In terms of mechanism, GPP underwent a methylation modification reaction at the C2 position by MT to form a cationic intermediate. Afterward, 2-methyl-GPP is generated by deprotonation, and 2-methylgeranyl cation is produced in the presence of MIBS. Then, 2-methyl linalyl diphosphate is formed as a result of isomerization. Additionally, the carbon chain of the 2-methyl linalyl cation is closed through reionization, leading to the formation of a 2-methyl terpinyl cation. Further cyclization results in the production of 2-methylbornyl cation. Finally, the capture of a water molecule leads to the formation of 2-MIB (Brock et al. 2013). The subsequent studies resolved the crystal structure of MIBS in *S. coelicolor* A3 (2). The resolutions of the unliganded-state MIBS with the coordination compounds of Mg^2+^ and 2-fluoroneryl diphosphate were 1.85 Å and 2.00 Å, respectively. MIBS can catalyze 2-methyl-GPP to undergo cyclization. In addition, the cyclization reaction can be completed by the diphosphate intermediate 2-methyllinalyl diphosphate following the sequence of ionization-isomerization-ionization (Köksal et al. 2013).

In most actinomycetes, the 2-MIB operon gene order is nucleotide-binding protein, MIBS, and GPPMT. However, in the cyanophyte *P. limnetica*, the 2-MIB operon gene order is reversed. Despite this difference, the 2-MIB synthesis pathway remains similar in both, with GPP being methylated GPPMT to produce 2-methyl-GPP and subsequently cyclized by MIBS to produce 2-MIB (Giglio et al. 2011). In the terpene cyclization of Pfl_1841, a terpene cyclase of *Pseudomonas fluorescens* origin, 2-MB is generated instead of 2-MIB (Fig. 1). This involves deprotonation at the C2 position of the carbonium ion intermediate, with no water molecule trapping at the final stage, leading to the production of 2-MB (Dickschat 2016). The introduction of 2-MB synthase (MBS) from PfO-1 and *M. olivasterospora*, as well as four genes (including MIBS) from *S. griseus* and *S. coelicolor* into 2-methyl-GPP-producing *E. coli*, ultimately generates different terpene products, including 2M2B, 1MC, 2-methylmyrcene, 2-methyllimonene, 2-methyl-β-fenchol, 2-methyl linalool, 2-methyl-α-terpineol, 2-methyl geraniol, and 2-methyl nerol (Kschowak et al. 2018).

#### Biosynthesis of geosmin

The biosynthetic pathway of geosmin has been elucidated by isotope-labeled precursor feeding experiments, identification of geosmin synthase (GS), and characterization of its byproducts. Geosmin biosynthesis begins with the cyclization of FPP into germacradienol catalyzed by GS. The subsequent step involves the fracture of the retro-Prins, which leads to the deletion of acetone. Further cyclization forms octalin, which subsequently undergoes deprotonation and electron rearrangement. The final step involves the trapping of a water molecule to form geosmin (Cane et al. 2006; Dickschat et al. 2005; Jiang et al. 2007; Jiang and Cane 2008; Nawrath et al. 2008).

In another report, 262 identified candidate TPSs of bacterial origin were engineered into the fungi strain *Streptomyces avermitilis*, and the majority of enzymes were found to belong to sesquiterpene synthases, and the major products were sesquiterpenoids such as geosmin and epi-isozizaene. In addition, GS was present in the majority of actinomycetes (Yamada et al. 2015).

Protein analysis shows that GS is composed of two structural domains, the N-terminal and C-terminal domains. The N-terminal domain possesses terpene cyclase activity and enables the generation of germacradienol from FPP. In addition, the C-terminal structural domain catalyzes the generation of geosmin from germacradienol (Cane and Watt 2003; Gust et al. 2003; Jiang et al. 2007). Furthermore, the biosynthesis of FPP to geosmin is catalyzed by a single enzyme without the intervention of any other enzyme or the need for any redox cofactor (Jiang et al. 2006) (Fig. 1).

### Biosynthesis of noncanonical terpenoids with C16 carbon skeleton

#### Biosynthesis of homoterpenes

**Fig. 2:**
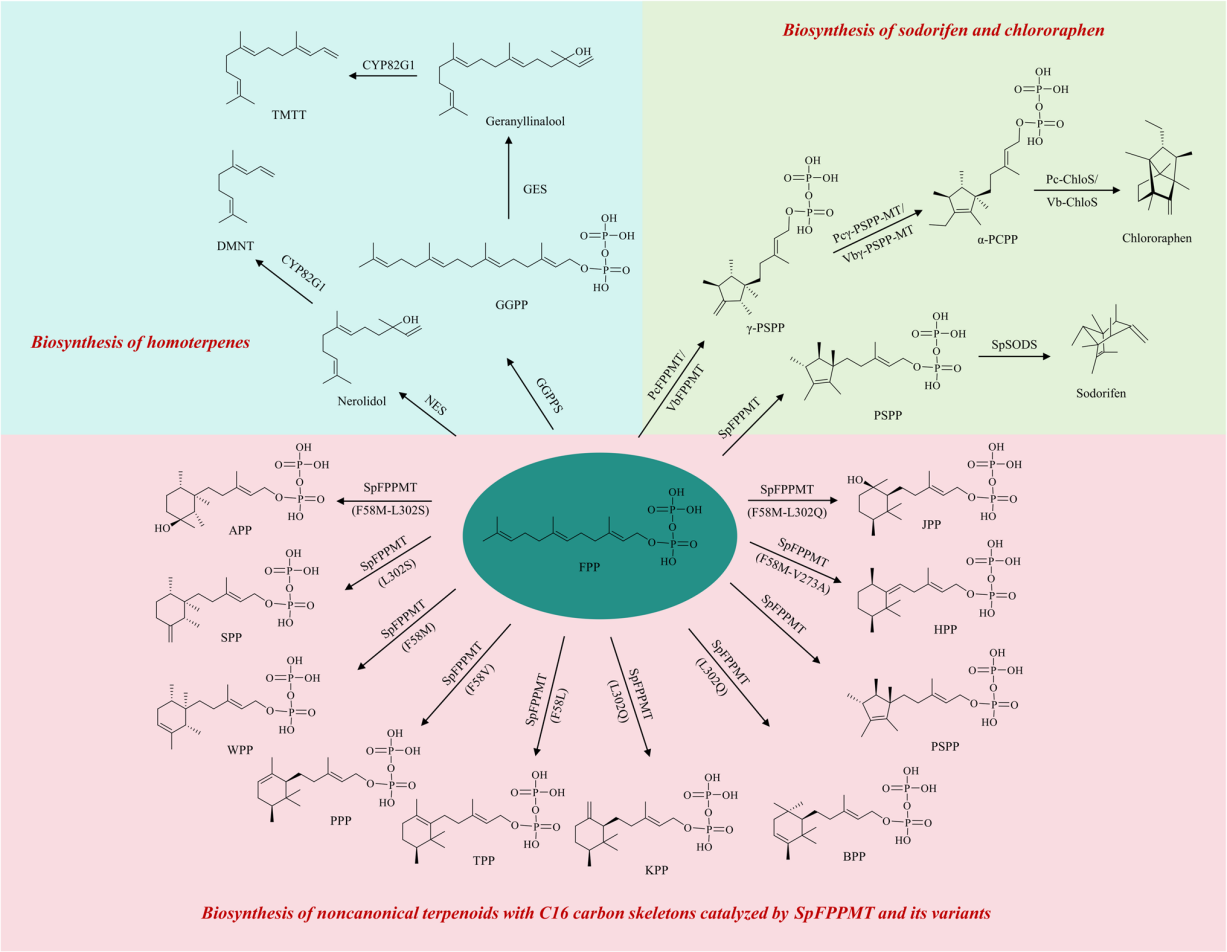
The biosynthesis of homoterpenes and other noncanonical terpenoids with C16 carbon skeletons. GGPP geranylgeranyl pyrophosphate, GGPPS GGPP synthase, GES geranyllinalool synthase, DMNT (*E*)-4,8-dimethyl-1,3,7-nonatriene, NES nerolidol synthase, TMTT (*E,E*)-4,8,12-trimethyltrideca-1,3,7,11-tetraene, PSPP presodorifen pyrophosphate, SpFPPMT FPP methyltransferase from *S. plymuthica*, SpSODS sodorifen synthase from *S. plymuthica*, PcFPPMT FPP methyltransferase from *P. chlororaphis*, VbFPPMT FPP methyltransferase from* V. boronicumulans*, Pcγ-PSPP-MT γ- PSPP methyltransferase from *P. chlororaphis*, Vbγ-PSPP-MT γ- PSPP methyltransferase from* V. boronicumulans*, α-PCPP α-prechlororaphen pyrophosphate, Pc-ChloS chlororaphen synthases from *P. chlororaphis*, Vb-ChloS chlororaphen synthases from* V. boronicumulans*, APP ancheryl diphosphate, SPP serratinyl diphosphate, WPP weylandtenyl diphosphate, PPP plymuthenyl diphosphate, TPP thorvaldsenyl diphosphate, KPP kimlarsenyl diphosphate, BPP blixenyl diphosphate, HPP hammershoyl diphosphate, JPP jacobsenyl diphosphate

During the biosynthesis of the homoterpene TMTT and its C11-analog DMNT (As DMNT is the homoterpene of TMTT, we put this C11-analog here for description), GGPP and FPP were catalyzed by geranyllinalool synthase (GES) and nerolidol synthase (NES), respectively, to first generate geranyllinalool and nerolidol. Subsequent isotope-labeling precursor experiments revealed that both geranyl linalool and nerolidol undergo oxidative degradation to ultimately produce their respective homoterpenes. In addition, a CYP450 enzyme, CYP82G1, found in *Arabidopsis*, comes into action (Fig. 2). In the process of TMTT and DMNT synthesis, the CYP82G1 catalyzes the degradation of homoterpenes to generate TMTT and DMNT, respectively (Lee et al. 2010). Meanwhile, two CYP450 enzyme genes, GhCYP82L1 and GhCYP82L2, were identified from *Gossypium hirsutum,* which has opened new insights into the biosynthesis of TMTT and DMNT. Heterologous expression in yeast and the subsequent enzyme analyses demonstrated that they are involved in the biosynthesis of TMTT and DMNT and can catalyze the conversion of geranyl linalool to DMTT or nerolidol to TMTT (Liu et al. 2018). DMNT-rich plants showcase significant potetial in “push-pull” strategies for pest management, offering an effective means to regulate insect behavior. For instance, transgenic tobacco co-expressing GhCYP82Ls and GhTPS14 can release DMNT in response to cotton bollworm attack, and the DMNT-releasing transgenic tobacco exhibit significant capability to attract the parasitoid wasp *Microplitis* mediator (Liu et al. 2021).

The control of pests and diseases in rice is particularly important as rice is a critical crop and a primary food for many. However, the biosynthetic pathway of homoterpene in rice is still unknown. The practical application of this indirect defense system also suffers from inherent limitations due to the quantitative constraints of homoterpene. However, recent in vitro biochemical functional characterization of yeast has discovered that OsCYP92C21 protein plays a pivotal role in the conversion of nerolidol and geranyl linalool to DMNT and TMTT, respectively. In addition, specific subcellular targeted expression, genetic transformation, and gene introgression have been reported to significantly increase the biosynthesis levels of DMNT and TMTT in rice. Thus, higher amounts of homoterpene can also be emitted even in the absence of inducing factors (Li et al. 2021).

#### Biosynthesis of sodorifen

Sodorifen, a novel 1,2,4,5,6,7,8-heptamethyl-3-methylene-bicyclo[3.2.1]oct-6-ene, is a major constituent of new hydrocarbons released by the rhizobium *Serratia odorifera* (von Reuss et al. 2010). FPP undergoes a SAM-dependent methylation modification reaction in the presence of SpFPPMT, an FPP methyltransferase from *S. plymuthica*, to generate presodorifen pyrophosphate (PSPP). During this process, the intermediate cyclohexyl carbocation does not undergo deprotonation as in typical terpene biosynthesis. Instead, it undergoes cyclization to generate an additional cycle. This step is catalyzed by the rare cyclase activity of SpFPPMT. PSPP, now containing a pentamethylcyclopentenyl group, undergoes further cyclization in the presence of *S. plymuthica* sodorifen synthase (SpSODS) to generate sodorifen (von Reuss et al. 2018) (Fig. 2). Moreover, 38 biosynthetic gene clusters (BGCs) were obtained by mining the *S. plymuthica* genome and through antiSMASH analysis. Using direct pathway cloning (DiPaC), 4.6 kb sodorifen candidate BGCs were successfully intercepted. Integrating them with the tetracycline-induced PtetO promoter and transforming them into *E. coli* resulted in the generation of large amounts of sodorifen in *E. coli* (Duell et al. 2019).

Apart from the rhizobium *S. plymuthica*, FPPMT was also identified in γ-proteobacterium *P. chlororaphis* O6 and β-proteobacterium *Variovorax boronicumulans* PHE5-4. In addition, methylation modification of PSPP by PSPPMT has been confirmed, revealing an noncanonical biosynthetic pathway for the first natural brexane-type bishomosesquiterpene, chlororaphen (C_17_H_28_). In the presence of enzymes like PcFPPMT or VbFPPMT, FPP is methylated to produce γ-presodorifen pyrophosphate (γ-PSPP, C16). Subsequent c-methylation of γ-PSPP by a second methyltransferase, Pcγ-PSPP-MT or Vbγ-PSPP-MT, produced α-prechlororaphen pyrophosphate (α-PCPP, C17). Finally, chlororaphen is generated under the catalysis of chlororaphen synthases (Pc-ChloS and Vb-ChloS) (Magnus et al. 2023) (Fig. 2). Bacterial genomic information mining led to the discovery of the sodorifen biosynthesis gene cluster. Subsequently, the TPSs from this gene cluster were extracted and introduced into an engineered yeast strain co-expressing SpFPPMT for fermentation. The research identified 47 different C16 terpenes in the products. Moreover, the structures of 13 different C16 noncanonical terpenes were resolved, highlighting the extensive structural diversity within this group of compounds (Duan et al. 2023).

### Biosynthesis of other noncanonical terpenoids with C16 carbon skeleton

Key amino acid residue sites in SpFPPMT are essential for its role. Disruption of the carbocation substrate through potential stabilization/destabilization of the carbocation intermediate, or through spatial interference can lead to no further generation of PSPP, but rather to the generation of other C16 backbones. Mutations (F58V, F58L, F58M) in the F58 site of SpFPPMT generated diphosphate building blocks such as plymuthenyl diphosphate (PPP), thorvaldsenyl diphosphate (TPP), and weylandtenyl diphosphate (WPP) in *S. cerevisiae*. Additionally, the L302 variant of SpFPPMT generated diphosphate building blocks such as blixenyl diphosphate (BPP), kimlarsenyl diphosphate (KPP), and serratinyl diphosphate (SPP). In addition, diphosphate building blocks such as jacobsenyl diphosphate (JPP), hammershoyl diphosphate (HPP), and ancheryl diphosphate (APP) were identified in the variants that had two mutated residues like SpFPPMT (F58M-L302S), SpFPPMT (F58M-L302Q), and SpFPPMT (F58M-V273A) (Fig. 2). These novel diphosphate building blocks when further processed with CYP450 enzymes ultimately led to the synthesis of 28 distinct C16 noncanonical terpenoids (Ignea et al. 2022).

In addition to its role in catalyzing the generation of 6-methyl-GPP, the GPPMT BezA can also undergo targeted mutation to obtain BezA (W210A), a mutant of BezA. W210A exhibits FPP C6 methyltransferase activity. In addition, this mutant enzyme can carry out a methylation reaction at the C6 position, effectively producing 6-methyl farnesyl pyrophosphate (6-methyl-FPP) using FPP as its substrate (Tsutsumi et al. 2022).

Excess methyl groups in propionyl-CoA in the LMVA pathway can be retained, culminating in the formation of noncanonical terpenoids of C16, C17, and C18. The introduction of MVA pathway genes from *Bombyx mori*, FPP synthase (FPPS) genes CfFPPS1 and CfFPPS2 from *Choristoneura fumiferana*, and epi-isozizaene synthase into *E. coli* ultimately generated noncanonical terpenoids of C16 as well as C17 and C18 compounds (Eiben et al. 2019).

#### Noncanonical triterpenoids synthesized from non-squalene substrates

It is generally accepted that triterpenoids are generated by triterpene synthase cyclization with squalene and 2,3-oxidosqualene as precursors (Abe 2007). However, terpenoids such as C10, C15, and C20 are short-chain terpenoids generated by cyclization, hydroxylation, and oxidation relying on polyisoprenyl diphosphates (Degenhardt et al. 2009; Minami et al. 2018). IPP and DMAPP are catalyzed by FPS to generate FPP. The two FPPs are then subjected to SS- and SE-catalyzed condensation to sequentially generate squalene and 2,3-oxidosqualene. These intermediates, squalene and 2,3-oxidosqualene, are subsequently cyclized by triterpene synthases (TrTSs) to form lanosterol, which is then modified by numerous enzymes to form desired triterpene compounds (Chen et al. 2023; Garcia-Bermudez et al. 2019). TPSs can be classified into two types, type I and type II, based on the amino acid conserved site and the mode of substrate protonation during catalysis. Type II terpene synthases catalyze subsequent reactions by protonating the substrate through a conserved “DXDD” motif, such as Squalene-Hopene Cyclase (SHC) and Oxidosqualene Cyclase (OSC). However, type I terpene synthases catalyze the subsequent reactions by binding to Mg^2+^ via the “DDXX(D/E)” and “NSE/DTE” domains and removing the substrate pyrophosphate group, such as methylisoborneol synthase and pentalenene synthase (Christianson 2017). Although type I terpene synthases are capable of synthesizing monoterpenes, sesquiterpenes, diterpenes, and dibenzoterpenes using isopentenyl pyrophosphate of different chain lengths as substrates, the synthesis of triterpenes using hexaprenyl pyrophosphate (HexPP, C30) as a substrate has not been reported yet. Moreover, HexPP is generated through HexPP synthase catalyzation by adding three IPP molecules to FPP following the C15-C20-C25-C30 sequence. It is important to note that HexPP synthase cannot synthesize GPP or FPP using IPP and DMAPP as substrates (Ogura et al. 1997; Sasaki et al. 2011). Furthermore, research on HexPP synthase (HexPPS) from *Sulfolobus solfataricus* has revealed that these can add IPP based on GGPP to generate HexPP. In addition, key amino acid residue sites determining the carbon chain length of the product have been identified by crystal structural analysis and site-directed mutagenesis (Sun et al. 2005).

In recent research, two bifunctional chimeric TPSs, TvTS and MpMS, were identified in the filamentous fungi *Talaromyces verruculosus* TS63-9 and *Macrophomina phaseolina* MS6, in which the synthesis of triterpenoids using HexPP as a substrate was realized for the first time. TvTS and MpMS can synthesize novel triterpenoid skeleton compounds, talaropentaene, and macrophomene, using either IPP and DMAPP or HexPP directly as substrates. This breakthrough discovery destroys the stereotype that triterpene skeletons can only be synthesized using squalene as a starting unit (Courdavault and Papon 2022). In vitro reaction and homologous activation experiments demonstrated that synthesizing triterpene skeletons is the inherent ability of TvTS and MpMS. In-depth investigation, including in vitro isotope feeding experiments resolved the absolute configuration of the products and the cyclization mechanism. For talaropentaene, cyclization initiates with a C1-III-IV reaction triggered by the removal of the HexPP pyrophosphate group. The ensuing 1,2-hydrogen ion migration and deprotonation culminate in the formation of talaropentaene. Macrophomene cyclization, however, commences with the departure of the HexPP pyrophosphate group, which triggers cyclization at the C1 and C22 positions. Deprotonation at the C1 position forms a ternary ring and ultimately macrophomene. To further investigate the generality of this synthesis and to precisely and efficiently target TPSs, research efforts expanded to include a wider range of natural triterpene species. Subsequently, batch prediction and molecular docking of TPS 3D structures were performed by AlphaFold2. As a result, two additional triterpene synthase genes, Cgl13855 and PTTC074, were successfully predicted and obtained. Both of them demonstrated the capability to generate the new triterpene colleterpenol. These results illustrate the generalizability of type I chimeric TPSs in catalyzing the cyclization of HexPP to produce triterpene skeletons (Tao et al. 2022).

### Nortriterpenes in* G. lucidum*

Ganoderma triterpenoids (GTs) are one of the main chemical components isolated from *G. lucidum* (Galappaththi et al. 2022). These triterpenoid compounds have complex and variable structures, and can undergo carbon reduction, ring opening, or rearrangement. Based on the number of carbon atoms in the skeleton, GTs are divided into three types, namely C30, C27, and C24-type (Gong et al. 2019). Among them, three C27-type nortriterpenoids, including lucidenic acid A, B, and C, were first discovered, and subsequently, C24-type nortriterpenoid compounds were identified, such as lucidones A, lucidones B, lucidones C, and lucidones H (Galappaththi et al. 2022). Compared with C30-type triterpenoids in *G. lucidum*, these C27- and C24-type nortriterpenoids are derived from lanostane-type triterpenoids with degraded side chains. However, little is known about the biosynthesis of C27-type and C24-type nortriterpenoids from lanosterol. Previous report has speculated the biosynthesis of one C24-type nortriterpenoid. The precursors ganoderic acid and its esterified derivatives underwent oxidation to form intermediates (such as lucidone A and lucidone D), which were further converted into lucidone J and lucidone K through addition reactions. Then, the compound lucidone K generated the C24-type nortriterpenoid lucidone I through elimination and addition reactions (Chen et al. 2017).

## Conclusion and prospects

The distinctive structures and activities of noncanonical terpenoids have attracted extensive attention. The current approaches for the biosynthesis of noncanonical terpenoids primarily involve two strategies: (1) The biosynthesis of noncanonical terpenoids can be achieved by harnessing specific methyltransferases that utilize IPP, GPP, and FPP as starting substrates. Alternatively, protein engineering techniques can be employed to modify these methyltransferases, enabling the synthesis of noncanonical terpenoids with varying chain lengths, such as C6, C7, C11, C12, and C16; this approach expands the diversity of terpene skeletons; and (2) Triterpenoids can be synthesized without the need for squalene as a substrate. This innovative approach enhances our understanding of the biosynthesis of terpenes in biological systems. Analyzing the biosynthetic pathways of noncanonical terpenoids and studying the functions of related enzymes allow access to a large number of key intermediates and elucidation of rare catalytic mechanisms. These discoveries serve as a robust foundation for subsequent large-scale preparation of these noncanonical terpenoids using synthetic biology and the exploration of new noncanonical terpenoids.

Future research on noncanonical terpenoids will be devoted to the identification of novel carbon skeletons and catalytic mechanisms, and the discovery of their bioactivities, with a focus on the following prospects: (1) Comprehensively analyzing the genomes of microorganisms and plants using high-throughput sequencing technology can excavate novel catalytic enzymes and biosynthetic pathways, facilitating the discovery of new types of noncanonical terpenoids; (2) Achieving the refined and modular design of biosynthetic pathways for noncanonical terpenoids and in-depth analysis of natural synthetic pathways can lead to the discovery of more efficient and controllable synthetic routes to realize the precise synthesis of target products; (3) Increased research on the biological activities and pharmacological effects of noncanonical terpenoids can fully stimulate their antimicrobial, antitumor, and antioxidant effects; (4) Integration of cutting-edge technologies, such as big data, artificial intelligence, and machine learning, can enable more accurate prediction and verification of biosynthetic pathways of noncanonical terpenoids.
